# Investigating the Therapeutic Effects of Albendazole, Mebendazole, and Praziquantel Nanocapsules in Hydatid Cyst-Infected Mice

**DOI:** 10.3390/pathogens14030240

**Published:** 2025-03-01

**Authors:** Nooshinmehr Soleymani, Soheil Sadr, Cinzia Santucciu, Abbas Rahdar, Giovanna Masala, Hassan Borji

**Affiliations:** 1 Department of Pathobiology, Faculty of Veterinary Medicine, Ferdowsi University of Mashhad, Mashhad P.O. Box 9177948974, Iran; nooshinmehrsoleymani@gmail.com (N.S.); soheil.sadr42@gmail.com (S.S.); 2 WOAH and NRL for Echinococcosis, Animal Health, Istituto Zooprofilattico Sperimentale della Sardegna, 07100 Sassari, Italy; giovanna.masala@izs-sardegna.it; 3 Department of Physics, University of Zabol, Zabol P.O. Box 53898615, Iran

**Keywords:** cystic echinococcosis, albendazole, hydatid cyst, mebendazole, mice, nanocapsules, praziquantel

## Abstract

Drug resistance is the main challenge in treating parasitic diseases, including cystic echinococcosis (CE). Hence, the current study aims to investigate the effect of nanocapsules containing albendazole (ABZ), mebendazole (MBZ), and praziquantel (PZQ) on treating hydatid cysts in mice using these high-potency drugs. A total of 78 female white laboratory mice (BALB/C mice), 8 weeks old and weighing 25 g, were intraperitoneally injected with 1500 live protoscoleces of *Echinococcus granulosus*. The first group received ABZ nanocapsules, group 2 received MBZ nanocapsules, group 3 received PZQ nanocapsules, group 4 received ABZ + MBZ nanocapsules, group 5 received ABZ + PZQ nanocapsules, and group 6 received MBZ + PZQ nanocapsules. Each group also had a control group, which received the non-nanocapsulated drugs (group 7–12). Group 13 received no treatment and served as the negative control, just receiving phosphate-buffered saline (PBS). A thorough examination of the cysts’ physical properties, including size, quantity, and weight, was carried out. According to our results, the polymeric nanocapsules are sphere-like and of different sizes. The total number of cysts in all nanocapsule groups significantly decreased compared to the control group. In the total weight of the cysts, ABZ + MBZ nanocapsules, ABZ + PZQ nanocapsules, and MBZ + PZQ nanocapsules had the least total cyst weight, showing that the use of the medicinal combination had a better effect on the penetration and weight reduction of the cysts. In conclusion, the findings showed that ABZ, MBZ, and PZQ significantly reduced the size, weight, and number of hydatid cysts in the mouse model used in this study.

## 1. Introduction

Cystic echinococcosis (CE) is a parasitic infectious disease caused by the larval stage of the parasite *Echinococcus granulosus sensu lato (s.l.)* belonging to the class Cestoda and family Taeniidae [1,2,3]. There are several ways in which CE is spread between the definitive and the intermediate hosts in the tapeworm’s life cycle [4]. Dogs and other carnivores, as definitive hosts of CE, harbor the adult worm in their small intestines, and the eggs are released in their feces [5]. Several intermediate hosts, such as sheep, goats, cattle, other herbivores, and humans as aberrant hosts, can become infected following the consumption of vegetables contaminated with dog feces containing parasite eggs [6,7,8]. The larvae hatch from the eggs and then migrate through the intestine to the bloodstream, forming hydatid cysts on various organs, such as the liver, lungs, and sometimes even brain and bones [9,10,11].

Cystic echinococcosis is a significant zoonotic disease due to its heavy burden on human and animal health and the economic losses resulting from the disease [12,13,14,15]. CE is more prevalent in rural areas and communities where dogs and sheep interact closely. Several regions, such as South America, Africa, the Middle East, Europe, and Central Asia, have a high incidence of CE [16,17,18,19]. It has been reported that CE is one of the most critical health and veterinary problems worldwide [20,21,22,23,24].

The CE diagnosis is often challenging since clinical signs and symptoms are manifested only after several years when the cyst increases in size and compresses the surrounding organs. Diagnosis is mainly based on imaging techniques, such as ultrasound (US) and computed tomography (CT) scans [25,26]. In addition, serological tests are available to detect anti-echinococcal antibodies in a patient’s blood, such as enzyme-linked immunosorbent assay (ELISA), immunochromatography test (ICT), and immunoblotting (IB) [27,28]. However, it may be necessary to combine different diagnostic methods for a solid and definitive diagnosis of CE [29]. Moreover, direct analysis of the parasitic material, such as parasitological, histological, and molecular biology analyses, provide reliable results to make a correct diagnosis, but it can be performed only after a very invasive approach, the surgical removal of the cystic lesion, which is only used as a save-life treatment. A less invasive approach is to watch and wait for the percutaneous aspiration (PAIR) of the cyst. However, any clinical protocol or specific treatment chosen for the management of the patient always involves the administration of antiparasitic drugs, which are usually a good cure option for hydatid cysts [30].

Among the chemical drugs used to treat CE, albendazole (ABZ), mebendazole (MBZ), and praziquantel (PZQ) are the most commonly used, and the mechanism of action of PZQ on cestodes primarily consists of increasing the permeability of their cell membranes to calcium ions (Ca^2+^) [31,32,33,34,35]. Since PZQ was considered a broad-spectrum anthelmintic in 1975, several studies have reported its successful use in the treatment of most diseases caused by trematodes and cestodes that affect humans and farm animals [36]. These infections include schistosomiasis, clonorchiasis, opisthorchiasis, paragonimiasis, heterofidiasis, echinostoisasis, fasciolopsiasis, neodiplostomiasis, gymnophaloidiasis, taeniasis, diphylobotriasis, hyponolepiasis, and cyanolepiasis. However, there could be many complications associated with the pharmacological treatment of hydatid cysts. It is usually necessary to use these drugs for long periods, and they may cause serious side effects [37]. Moreover, several challenges are associated with this treatment of CE, such as their thick walls, complex structures, and difficulty in enabling drugs to penetrate these cysts.

Nanotechnology encompasses understanding nanometer-scale objects’ fundamental physics, chemistry, biology, and technology [38]. Nanoparticles (NPs) have several unique properties that enable them to be applied in various medical, industrial, and other fields [39]. One of the potential uses of nanotechnology in medicine is in developing targeted drug delivery systems capable of delivering drugs directly to the site of infection or tumor to enhance the effectiveness of the drugs. CE can be effectively treated using nanotechnology with antiparasitic medications and various nanotechnology applications [40]. Using nanocapsules as one of the new drug delivery systems can allow drugs to be directly and more efficiently delivered to the infection site of the parasites [41]. Using nanocapsules coated with polymers and lipids, drugs can be protected from degradation and released gradually over time. Hence, the drugs can reach the parasites more effectively, and the treatment can be more effective. In vivo studies of the effects of nanoparticles on CE provide valuable information on the potential of this technology to improve the treatment of parasitic diseases (Table 1).

The current study investigates the therapeutic effect of nanocapsules coated with different drugs, ABZ, MBZ, and PZQ, on hydatid cysts of experimentally infected CE mice.

## 2. Materials and Methods

### 2.1. Chemical Sources

Albendazole: Damyabendazol600, Damyaran Arak, Iran. Praziquantel: Lorencit 50, Damdaran Razak, Iran. Mebendazole: 100, ErfanDarou, Iran. Ethyl butyrate: Merck code 800500. Phosphate-buffered saline tablet SIGMA 4221. Ketamin 10%: Bermer Pharma, Warburg, 34414 Germany. Alfasan Xylazine 2%: Woerden, The Netherlands.

### 2.2. Nanocapsules Preparation

At room temperature, drug-loaded oil-in-water surfactant-based biocompatible nanomicelles were developed from dissolving drugs in 1% (*w*/*w*) solutions suitably diluted amount of fatty acid sodium caprylate (SC, 0.09 g) and F127 (0.009 g), phosphate-buffered saline (PBS at pH 7.4) under vigorous stirring at a fixed ethyl butyrate-to-surfactant molar ratio of 10 and final total volume of 50 mL. The excess of free PHT was eliminated by dialysis for 24 h [53].

### 2.3. Zeta, Scanning Electron Microscope, and Particle Size Analysis

Twenty-four hours before the zeta potential test, scanning electron microscope (SEM), and particle size analysis, ABZ, MBZ, and PZQ solutions were made with lipid nano-polymeric capsules according to the instructions. Then, each prepared sample was poured into separate microtubes, mixed with 10 µL of PBS, and transferred to the central laboratory. The particle size and size distribution of the nanoparticles obtained were assessed using a particle size analyzer (PSA; SHIMADZU, SALD-2101, Kyoto, Japan). Additionally, the measurements were reconfirmed at predetermined intervals. Zeta potential was determined using Zeta-Chek (Microtract, ZC007, Duesseldorf, Germany). Furthermore, for conducting the SEM, the samples were first prepared using the Sputter Coater (SC7620) Polaron Range, and finally, SEM was performed by LEO 1450VP.

Based on the first part of this study, previously performed on the in vitro evaluation of ABZ, MBZ, and PZQ nanocapsules on protoscoleces (PSCs) (https://doi.org/10.3390/pathogens13090790) [54], the zeta potential spectra of the nanocapsules loaded with ABZ range from 5 to −55 millivolts (mean: −35.78 mW), MBZ range from 0 to −48 millivolts (mean: −27.38 mW), and PZQ range from 0 to −43 millivolts (mean: −23.34 mW). Moreover, the mean size of ABZ nanocapsules is 193.01 nm, MBZ is 170.40 nm, and PZQ is 180.44 nm.

### 2.4. Protoscolex Collection

In this experimental protocol, PSCs were collected from sheep’s liver from the slaughterhouse of Mashhad, Iran. To ensure cleanliness, the PSCs were thoroughly washed with pure PBS (pH 7.2) before being placed into the automated system. Next, PSC viability was evaluated under a microscope by examining the movement of the worm parasites, followed by staining with an eosin solution at a concentration of 0.1% for further examination of PSC vitality [55,56]. Eosin was absorbed by dead PSCs, portraying them in a red hue, whereas unstained live PSCs were considered viable and colorless.

### 2.5. Mouse Treatments with Nanocapsule Drugs

A total of 78 female white laboratory mice (BALB/C mice), 8 weeks old and weighing 25 g, were intraperitoneally injected with 1500 live PSCs of *E. granulosus* (with a survival of 90%) and were divided randomly into 13 groups (*n* = 6). The infected mice were kept under correct conditions of light (12 h/12 h dark), humidity, and temperature for 7 months, and animals had free access to food and water ad libitum. After 7 months, the treatment began.

Treatment groups were as follows: group 1 received ABZ nanocapsules (Nano-ABZ); group 2 received MBZ nanocapsules (Nano-MBZ); group 3 received PZQ nanocapsules (Nano-PZQ); group 4 received ABZ + MBZ nanocapsules (Nano-ABZ + MBZ); group 5 received ABZ + PZQ nanocapsules (Nano-ABZ + PZQ); and group 6 received MBZ + PZQ nanocapsules (Nano-MBZ + PZQ). Each group also had a positive control group and received only chemical medicine, not nanomedicine (group 7–12). Group 13 received no treatment and served as the negative control group and just received PBS (Table 2).

The mice received the drugs orally using the gavage method for 60 days. The dose of the ABZ, MBZ, PZQ, ABZ + MBZ, ABZ + PZQ, and MBZ + PZQ nanocapsules was 1 mg/kg, with a concentration of 1 mg/mL and a medicine amount of 300 µL.

The dose of ABZ (control) and MBZ (control) was 150 mg/kg, PZQ (control) 600 mg/kg, ABZ + MBZ (control), 150 mg/kg + 150 mg/kg, ABZ + PZQ (control) 150 mg/kg + 600 mg/kg, and MBZ + PZQ (control) 150 mg/kg + 600 mg/kg, and the amount of medicine was 300 µL.

### 2.6. Parasitological Studies

**Figure 1:**
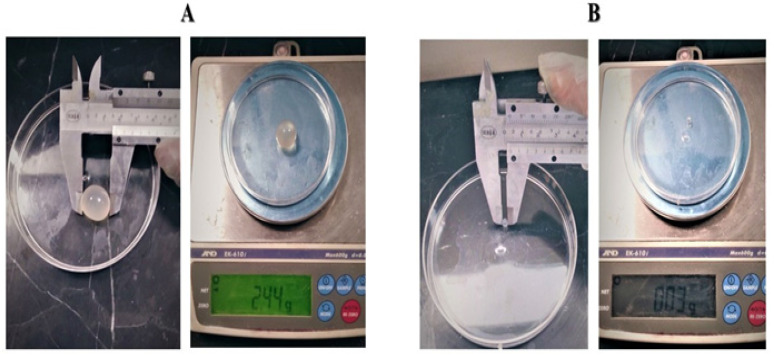
Measurement and weighing of cysts. (**A**) is an example of measuring and weighing a large cyst. (**B**) is an example of measuring and weighing a small cyst.

At the end of the experiments, corresponding to 60 days, mice of all groups were euthanized using 87.5 mg/mL ketamine + 12.5 mg/mL xylazine intraperitoneally (IP), according to the current legislation on animal welfare. Following euthanasia, cysts were observed in the abdominal cavity and then carefully collected along with the internal organs. A parasitological examination of the cysts was promptly performed, determining external features, including size, shape, quantity, and weight (Figure 1). Finally, the internal content of the cystic liquid was examined under a light microscope. To verify the success of the animal model, we included positive control groups (groups 7–12) treated with commercially available chemical drugs commonly used for routine treatment.

### 2.7. Statistical Analysis

SPSS software version 26 was used for statistical analysis in this study. The Mann–Whitney test and *t*-test were used to compare between groups. Furthermore, post hoc tests included Bonferroni and Tukey–Kramer. The statistical tests were conducted with significance levels of less than 0.05.

## 3. Results

### 3.1. Scanning Electron Microscope

**Figure 2:**
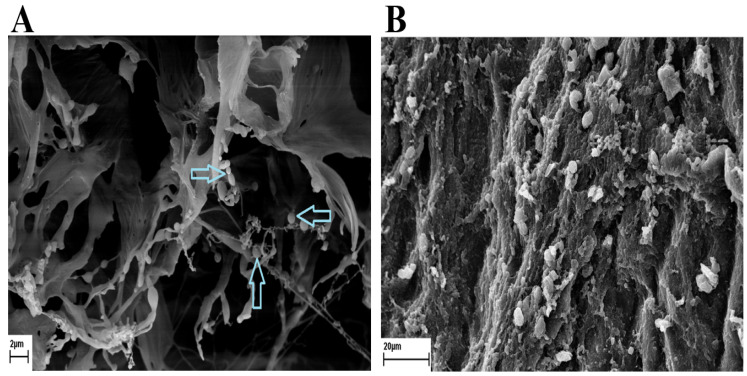
(**A**) The figure shows that the polymeric nanocapsules are shaped like spheres of different sizes. The blue arrows are pointing to the nanocapsules. (**B**) The SEM image of penetrating nanocapsules into the hydatid cyst wall.

According to the results, the figure shows that the polymeric nanocapsules are shaped like spheres of different sizes (Figure 2).

### 3.2. Mouse Treatment with Nanocapsule Drugs

During the study, there were no casualties in the nanocapsule groups, but ABZ had one, MBZ had one, PZQ had two, ABZ + MBZ had one, ABZ + PZQ had one, MBZ + PZQ had one, and finally, the control negative group had three casualties.

#### 3.2.1. Number of Cysts

The total number of cysts in all groups treated with nanocapsules coated with drugs significantly decreased compared to the control groups (*p* < 0.05).

**Figure 3:**
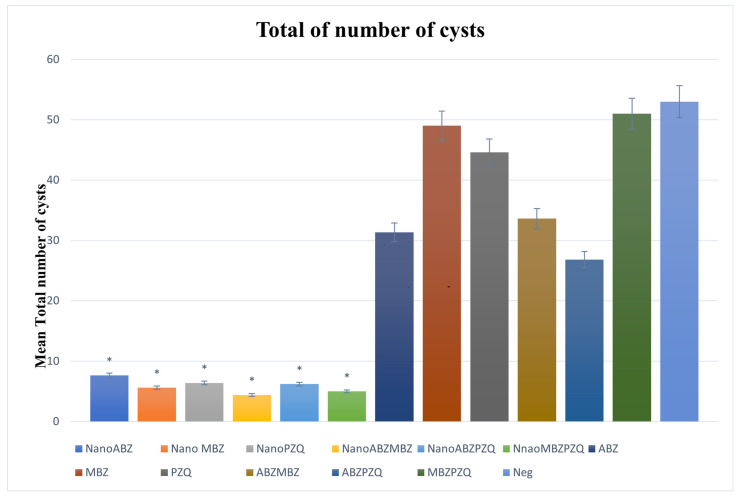
Analysis of the mean quantity of parasitic lesions presented by each group of mice treated with nanocapsules and their controls. The nanocapsule groups significantly decreased the number of cysts compared to their positive control and negative groups (*p* < 0.05). The asterisk (*) indicates significant differences between nanocapsules and conventional treatment groups.

In group ABZ nanocapsules, the number of cysts was significantly lower (*p* < 0.05) compared to the ABZ group. Moreover, in the MBZ nanocapsule group, the total number of cysts decreased significantly compared to MBZ. The ABZ and ABZ + MBZ groups had almost similar results, with no significant difference. However, when the two drugs were combined in the nanocapsules, there was a significant decrease in the total number of cysts compared to the ABZ and ABZ + MBZ groups (*p* < 0.05). Additionally, the number of cysts decreased in the group treated with PZQ, and even the behavioral side effects were much less in the mice treated with PZQ nanocapsules than in the PZQ group (Figure 3).

#### 3.2.2. Total Cysts Weight

The total weight of the cysts in all the treatment groups with nanocapsules was significantly reduced compared to their control groups.

**Figure 4:**
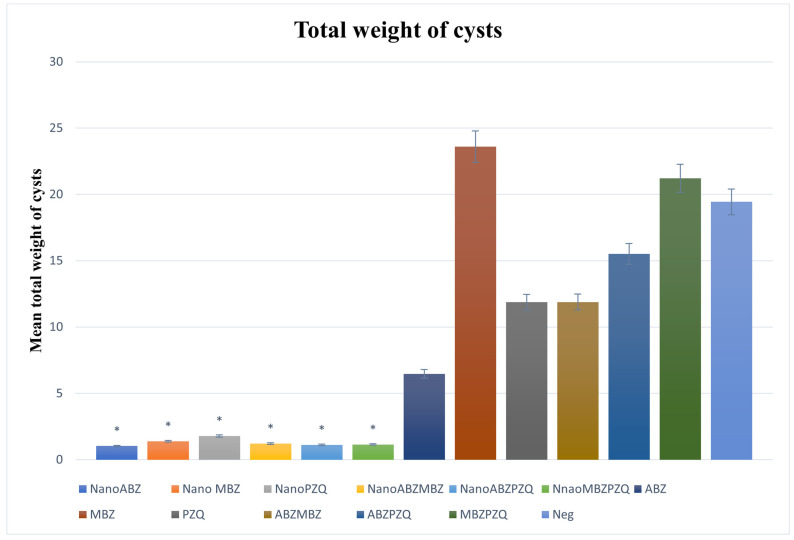
Analysis of the mean total weights of cysts in the nanocapsule groups and their positive control groups. Compared to control groups, nanocapsules significantly decreased the total weight of cysts (*p* < 0.05). The asterisk (*) indicates significant differences between nanocapsules and conventional treatment groups.

In the total weight of the cysts, ABZ + MBZ nanocapsules, ABZ + PZQ nanocapsules, and MBZ + PZQ nanocapsules had the least total cyst weight, showing that the use of the medicinal combination had a better effect on the penetration and weight reduction of the cysts. In the control group, it was observed that MBZ had no significant effect on reducing the weight of the cysts (Figure 4).

#### 3.2.3. Minimum Cyst Size

**Figure 5:**
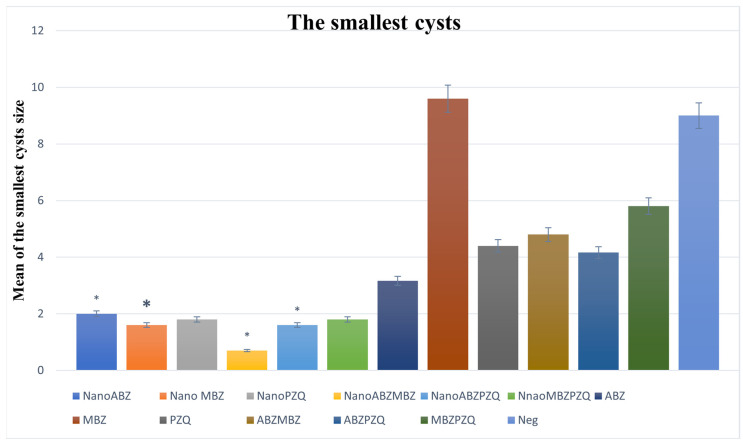
Comparison of cyst size and standard deviation (SD) between nanocapsules and control groups. The size of the smallest cysts decreased significantly in the nanocapsule group. ABZ nanocapsules and ABZ (*p* = 0.03). MBZ nanocapsules and MBZ (*p* = 0.001). PZQ nanocapsules and PZQ (*p* = 0.009). ABZ + MBZ nanocapsules and ABZ + MBZ (*p* = 0.001). ABZ + PZQ nanocapsules and ABZ + PZQ (*p* = 0.0005). MBZ + PZQ nanocapsules and MBZ + PZQ (*p* = 0.001). The asterisk (*) indicates significant differences between nanocapsules and conventional treatment groups.

The size of the smallest cysts decreased in all nanocapsule groups compared to the control groups, and the ABZ + MBZ nanocapsule group had the best result (Figure 5).

#### 3.2.4. Maximum Cyst Size

**Figure 6:**
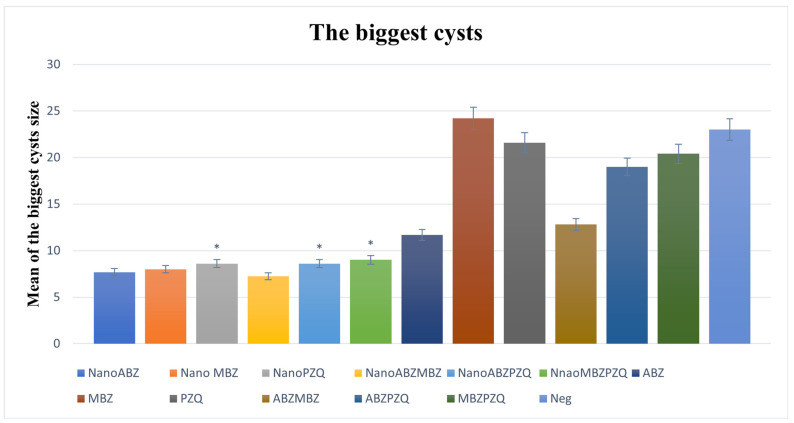
Comparison of cyst size and standard deviation (SD) between nanocapsules and control groups. All nanocapsule groups significantly reduced the size of the largest cyst compared to the control groups except for ABZ. ABZ nanocapsules and ABZ (*p* = 0.19). MBZ nanocapsules and MBZ (*p* = 0.01). PZQ nanocapsules and PZQ (*p* = 0.0006). ABZ + MBZ nanocapsules and ABZ + MBZ (*p* = 0.06). ABZ + PZQ nanocapsules and ABZ + PZQ (*p* = 0.001). MBZ + PZQ nanocapsules and MBZ + PZQ (*p* = 0.0006). The asterisk (*) indicates significant differences between nanocapsules and conventional treatment groups.

All nanocapsule groups significantly reduced the size of the largest cyst compared to the control groups except for ABZ, and the best result was related to the combination of ABZ + MBZ nanocapsules (Figure 6).

#### 3.2.5. Maximum Cyst Weight

**Figure 7:**
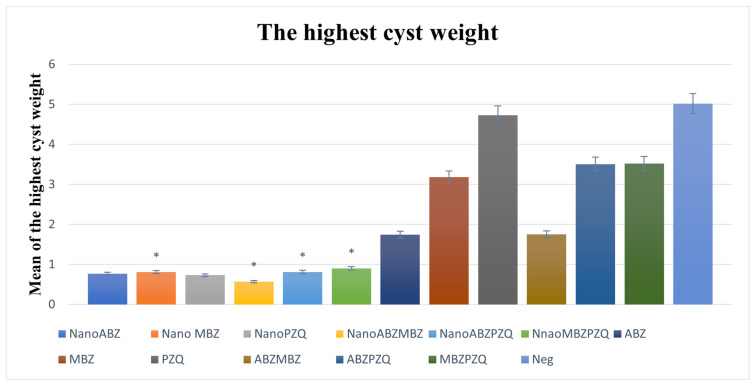
The weight of the heaviest cyst was significantly decreased in the nanocapsule groups compared to their control groups, except for ABZ. ABZ nanocapsules and ABZ (*p* = 0.19). MBZ nanocapsules and MBZ (*p* = 0.005). PZQ nanocapsules and PZQ (*p* = 0.001). ABZ + MBZ nanocapsules and ABZ + MBZ (*p* = 0.001). ABZ + PZQ nanocapsules and ABZ + PZQ (*p* = 0.005). MBZ + PZQ nanocapsules and MBZ + PZQ (*p* = 0.001). The asterisk (*) indicates significant differences between nanocapsules and conventional treatment groups.

The weight of the heaviest cyst was significantly decreased in the nanocapsule groups compared to their control groups, except for ABZ. In the examination of the weight of the largest cysts, the best result was in the ABZ + MBZ nanocapsule group, which shows that the effectiveness of MBZ increases on the side of ABZ. In the nanocapsule groups, ABZ nanocapsules, MBZ nanocapsules, and PZQ nanocapsules had almost the same weight loss in the largest cysts (Figure 7).

#### 3.2.6. Minimum Cyst Weight

**Figure 8:**
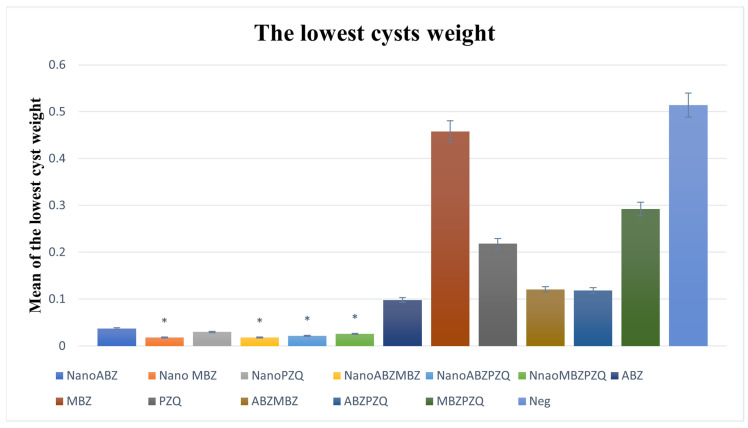
Comparison of cyst weight and standard deviation (SD) between nanocapsules and control groups. The lowest-weight cysts in the nanocapsule groups decreased significantly compared to the control groups, except for ABZ. ABZ nanocapsules and ABZ (*p* = 0.66). MBZ nanocapsules and MBZ (*p* = 0.002). PZQ nanocapsules and PZQ (*p* = 0.04). ABZ + MBZ nanocapsules and ABZ + MBZ (*p* = 0.02). ABZ + PZQ nanocapsules and ABZ + PZQ (*p* = 0.0003). MBZ + PZQ nanocapsules and MBZ + PZQ (*p* = 0.002). The asterisk (*) indicates significant differences between nanocapsules and conventional treatment groups.

**Figure 9:**
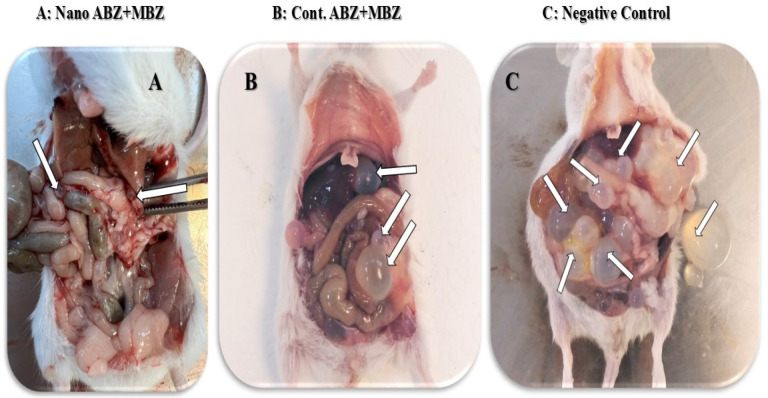
Mice after intraperitoneal treatment with protosceleces that display hydatid cysts in the abdominal cavity organs. (**A**) Group treated with ABZ + MBZ nanocapsules. Arrows show two cysts that are very small due to treatment. (**B**) Group treated with ABZ + MBZ as a positive control. (**C**) The negative control group was treated only with PBS. Reduction in the number and size of cysts in the nanocapsule groups compared to the negative control and positive control groups (with arrows pointing to the cysts).

The lowest-weight cysts in the nanocapsule groups decreased significantly compared to the control groups, except for ABZ (Figure 8 and Figure 9).

In evaluating the performance of nanocapsules in treatment groups compared to positive controls, the performance improvement rate of ABZ nanocapsules was 63.90% better than ABZ, MBZ nanocapsules were 85.78% better than MBZ, PZQ nanocapsules were 78.87% better than PZQ, ABZ + MBZ nanocapsules were 79.30% better than ABZ + MBZ, ABZ + PZQ nanocapsules were 76.73% better than ABZ + PZQ, and MBZ + PZQ nanocapsules were 82.48% better than MBZ + PZQ.

## 4. Discussion

Several conventional treatments are available for CE that could be more or less invasive, but for the correct patient management, all medical approaches always involve pharmacological treatment [57,58,59,60]. This is usually comprising antiparasitic drugs such as ABZ, MBZ, and PZQ [61,62]. However, there are several limitations to these medications, such as drug resistance and ineffective penetration into the inner layers of cysts, which has led researchers to explore the use of nanocapsules to improve the efficacy of these medications [63]. The main challenge in treating parasitic diseases, including hydatid cysts, is dealing with drug resistance. The misuse of antiparasitic drugs, as well as the long-term use of pharmaceutical drugs, has resulted in parasites becoming less sensitive to conventional drugs when used long-term. Although ABZ is a highly effective drug, there have recently been more reports regarding drug resistance against it, and the efficacy of ABZ has decreased [64,65,66,67].

Nanotechnology can provide targeted drug delivery systems and increase drug absorption by infected tissues [68]. Nanoencapsulated drugs are a very effective way to prevent drug resistance. This is because they improve the drug’s pharmacokinetic properties and increase its bioavailability [69]. The small size and targetability of nanocapsules allow for the precise delivery of drugs to the tissue or cells that are infected directly, thanks to their small size and targeted delivery formula [70,71]. As a result, it is possible to reduce the required dose of the drug and the number of side effects during treatment [72]. To determine their efficacy, ABZ, MBZ, and PZQ nanocapsules were administered to CE-infected mice in the current study. According to these results, active substances delivered through nanocapsules can reduce the size, weight, and number of cysts. This reduction was very significant compared to the groups receiving the non-nano form of the drug. Furthermore, ABZ nanocapsules showed a higher efficacy level than MBZ and PZQ. This is likely due to their specific chemical characteristics, which allow them to penetrate the cyst’s inner layers better and achieve better results. Based on the findings of these studies, it can be concluded that nanotechnology can be used effectively and efficiently to improve drug effectiveness and reduce drug resistance.

According to the results of our previous in vitro studies [41], nanotechnology has shown great promise in treating parasitic diseases, and their results are highly consistent with previous research. Similar results have been found in CE animal models using ABZ nanocapsules to reduce sores and cysts. Nanocapsules could penetrate the cysts’ outer layer, increasing the drug concentration in infected tissues. Moreover, several studies have compared the effectiveness of NPs and nanoencapsulated herbal medicines [44,73,74,75,76]. In addition, herbal medicines have fewer side effects and are widely available, and they can be a good option for treating parasitic diseases. However, some limitations limit their use because of low absorption rates and low stability during absorption. Through nanoencapsulation of these herbal compounds, these limitations can be overcome, and their efficacy can be improved [59].

This study produced promising results, but there is still a lack of knowledge in this field (Table 1). More studies have to be performed, and some limitations must be addressed. One of these limitations was the need for a more detailed investigation of the nanocapsule mechanism of action at the molecular level. Moreover, biochemical analysis could also help identify the efficacy of nanocapsules. Further studies must also examine whether these nanocapsules will have long-term adverse side effects. Furthermore, using other animal models or clinical studies on humans may also confirm the findings of the current studies. This may help in making this technology a standard treatment method. Future studies should address these aspects to understand this issue better. An investigation into the effects of drug nanocapsules on the body’s immune mechanisms and how the body’s defense mechanisms interact with the body’s immune system can help us better understand the effects of these drugs on the human body. It is also possible to increase the effectiveness of treatment by combining chemical drugs with plant-based drugs in nanocapsules.

## 5. Conclusions

In conclusion, the findings showed that ABZ, MBZ, and PZQ significantly reduced the size, weight, and number of hydatid cysts in the mouse model used in this study. These results are consistent with previous research and demonstrate nanotechnology’s unique benefits in treating parasitic diseases. However, further development of this technology and clinical studies are necessary for its widespread use.

## Figures and Tables

**Table 1:** A list of in vivo studies on the effects of nanoparticles against hydatid cysts.

Nanoparticles	Animal	Gender	Drug Dosage	Treatment Period	Result	References
Selenium nanoparticles(SeNPs)	Mus musculus BALB/c	male	protoscoleces processed with 50, 100, 150, 200 µg/mL Se NPs	3, 4, 5 months post infestation	The reduction rate was 90% in mice injected with exposed protoscoleces at 150 µg/mL in the next 4–5 months of infection.	S.A. Mohammed and A.A. Ali. (2022) [42]
Silver nanoparticles (AgNPs) accomplished using Zizyphus spina-christi leaves.	BALB/c	male and female	50, 100, 200, 300 mg/kg	30 days	Ag NPs did not induce any adverse effects or signs and no death. Change in the appearance of the liver hydatid cysts from hyaline to milky cloudy.	S.M. Hamad et al. (2022) [43]
Albendazole sulfoxide (ABZ-SO)-loaded Chitosan–PLGA	Laboratory mice	-	10 mg/kg	45 days at a daily dose	The weight and volume of cysts were statistically significant	M.M. Darvishi. et al. (2020) [44]
Sea urchin gonad extraction combined with Tio_2_ NPs	BALB/c mice	-	15 µg/mL	3 months	Reduction in number, size, and volume of the cysts.	A. Navvabi. et al. (2019) [45]
Holothuria leucospilota extract and CeO_2_ nanoparticles	BALB/c mice	male	50 mg/kg	1 month	Decrease in number of cysts, size, and volume of cyst.	S. Aryamand. et al. (2019) [46]
Albendazole-loaded silver nanoparticles	Laboratory-bred albino mice	female	200 mg/kg/d for 5 consecutive days per week	8 weeks	All the treated groups showed significant reductions in size and weight.	N.E. Nassef. et al. (2019) [47]
Albendazole LNCs	CF-1 mice	female	5 mg/kg/d	1 month	4 out of the 10 ABZ-LNC-treated mice did not develop any cysts. -Changes in the germinal layer.	G.V.U. Gamboa. et al. (2019) [48]
Chitosan–praziquantel and chitosan–albendazole nanoparticles	DBA/2 mice	male	25 mg/kg	21 days	Reduced the cyst numbers.	N. Torabi. et al. (2018) [41]
Flubendazole-loaded mPEG-PCL NPs	BALB/c mice	-	1, 5, 10 μg/mL/d	1 month	Reduced the weight and number of the cysts. (94.64% and 70.21%)	M. Farhadi. et al. (2018) [49]
Albendazole and praziquantel with loaded solid lipid nanoparticles components	BALB/c	female	50 mg/kg. 5 consecutive days and 2-day break every week	3-month treatment and 2-month rest	Reduced the wet weight and size of developed cysts (85%)	A. Jelowdar. et al. (2017) [50]
Albendazole -LNCs	CF-1 mice	female	5 mg/kg	1 month	Cysts from ABZ-LNC-treated mice were 1.7-fold higher than those detected in plasma (control).	P.E. Pensel. et al. (2015) [51]
Albendazole sulfoxide and albendazole sulfoxide-loaded solid lipid nanoparticles	Balb/c mice	-	0.5, 2 mg/kg/48 h	15 days	size and weight in the treated animals were reduced.	S. Ahmadnia. (2013) [52]

**Table 2:** Description of treatments with nanocapsules and their control for 13 groups of mice.

Groups	Treatments	Dose
n°	Nanocapsule Groups	
1	Nano-ABZ	1 mg/kg
2	Nano-MBZ	1 mg/kg
3	Nano-PZQ	1 mg/kg
4	Nano-ABZ + MBZ	1 mg/kg, (Both at 1 mg/mL final concentration), (Both ABZ + MBZ in the one nanocapsule)
5	Nano-ABZ + PZQ	1 mg/kg, (Both at 1 mg/mL final concentration), (Both ABZ + PZQ in the one nanocapsule)
6	Nano-MBZ + PZQ	1 mg/kg, (Both at 1 mg/mL final concentration), (Both MBZ + PZQ in the one nanocapsule)
	Positive control groups with only drugs	
7	ABZ	150 mg/kg
8	MBZ	150 mg/kg
9	PZQ	600 mg/kg
10	ABZ + MBZ	150 mg/kg + 150 mg/kg
11	ABZ + PZQ	150 mg/kg + 600 mg/kg
12	MBZ + PZQ	150 mg/kg + 600 mg/kg
	Negative Control group	
13	PBS	1 mg/mL

## Data Availability

The datasets generated during and/or analyzed during the current study are available from the corresponding author upon reasonable request.
